# Nanoparticles in Ocular Drug Delivery Systems

**DOI:** 10.3390/pharmaceutics15061675

**Published:** 2023-06-08

**Authors:** Hugo Almeida, Ana Catarina Silva

**Affiliations:** 1 UCIBIO (Research Unit on Applied Molecular Biosciences), REQUIMTE (Rede de Química e Tecnologia), MEDTECH (Medicines and Healthcare Products), Laboratory of Pharmaceutical Technology, Department of Drug Sciences, Faculty of Pharmacy, University of Porto, 4050-313 Porto, Portugal; 2 Associate Laboratory i4HB-Institute for Health and Bioeconomy, Faculty of Pharmacy, University of Porto, 4050-313 Porto, Portugal; 3 Mesosystem Investigação & Investimentos by Spinpark, Barco, 4805-017 Guimarães, Portugal; 4 FP-BHS (Biomedical and Health Sciences Research Unit), FP-I3ID (Instituto de Investigação, Inovação e Desenvolvimento), Faculty of Health Sciences, University Fernando Pessoa, 4249-004 Porto, Portugal

Conventional ophthalmic formulations lack a prolonged drug release effect and mucoadhesive properties, decreasing their residence time in the precorneal area and, therefore, in drug penetration across ocular tissues, presenting low bioavailability with a consequent reduction in therapeutic efficacy. These limitations are related to the physiological mechanisms of the eye. To increase the residence time in formulations on the surface of ocular tissues and increase their ability to penetrate these tissues, different strategies can be used, namely, the use of viscosifying agents, mucoadhesive polymers, stimuli-responsive polymers, microparticles, and colloidal carriers (e.g., micelles, liposomes, nanosuspensions, nanoemulsions, and polymeric and lipid nanoparticles).

We are delighted to present the latest research and review works reporting on the use of nanoparticles and other nanosystems in ophthalmic formulations to increase the bioavailability of drugs, improving their therapeutic efficacy. The articles selected for this Special Issue are the following:

1. “Development of Triamcinolone Acetonide Nanocrystals for Ocular Administration” [1].
2. “Design, Characterization and Pharmacokinetic–Pharmacodynamic Evaluation of Poloxamer and Kappa-Carrageenan-Based Dual-Responsive In Situ Gel of Nebivolol for Treatment of Open-Angle Glaucoma” [2].
3. “Cannabidiol Loaded Topical Ophthalmic Nanoemulsion Lowers Intraocular Pressure in Normotensive Dutch-Belted Rabbits” [3].
4. “Enhanced Ocular Anti-Aspergillus Activity of Tolnaftate Employing Novel Cosolvent-Modified Spanlastics: Formulation, Statistical Optimization, Kill Kinetics, Ex Vivo Trans-Corneal Permeation, In Vivo Histopathological and Susceptibility Study” [4].
5. “Photodisruption of the Inner Limiting Membrane: Exploring ICG Loaded Nanoparticles as Photosensitizers” [5].
6. “Linoleic Acid-Based Transferosomes for Topical Ocular Delivery of Cyclosporine A” [6].
7. “Ciprofloxacin-Loaded Zein/Hyaluronic Acid Nanoparticles for Ocular Mucosa Delivery” [7].
8. “Physicochemical and Stability Evaluation of Topical Niosomal Encapsulating Fosinopril/γ-Cyclodextrin Complex for Ocular Delivery” [8].
9. “Recent Progress in Chitosan-Based Nanomedicine for Its Ocular Application in Glaucoma” [9].
10. “Ocular Delivery of Therapeutic Proteins: A Review” [10].
11. “Bioprinted Membranes for Corneal Tissue Engineering: A Review” [11].
12. “Fluorescent Nanosystems for Drug Tracking and Theranostics: Recent Applications in the Ocular Field” [12].

The selected articles highlight the use of nanotechnology in ophthalmic formulations to encapsulate molecules with different therapeutic applications, including antibiotics, anti-inflammatory drugs, natural compounds, and proteins. The use of nanosystems in different approaches, such as theranostics and nanobubbles, is also presented, showing their potential for ocular application.

We would like to take this opportunity to thank all the authors and reviewers for their incredible work. Without their contribution, it would not be possible to publish this Special Issue with such high scientific quality. We hope that the readers of this Special Issue enjoy these articles, and that they will serve as a starting point for further research and/or review articles, thus contributing to scientific discovery and progress in this field.
